# Identification of Cyclospora in poultry.

**DOI:** 10.3201/eid0204.960414

**Published:** 1996

**Authors:** H L García-López, L E Rodríguez-Tovar, C E Medina-De la Garza


coordinating system among agencies at all levels and deal with the threat of widespread, multistate/international foodborne outbreaks caused by infectious or toxic agents.

**Daniel G. Colley**
Centers for Disease Control and Prevention, Atlanta, Georgia, USA

### Identification of Cyclospora in Poultry

**To the Editor:** Human infection with the parasitic protozoa, *Cyclospora*, was first described in 1979 (1), and the organism was only recently categorized as an important gastrointestinal parasite. A single species, *Cyclospora cayetanensis*, has been described in humans (2), while most species in the genus *Cyclospora* have been described only in reptiles and rodents (3). The consumption of undercooked meat and exposure to contaminated water have been considered possible sources of human infection with *C. cayetanensis* (1,4). Coccidia were detected in drinking water in Nepal (5), and the parasite was identified in an animal species (one duck in Peru, by Zerpa et al. [6]) different from those in which it was described earlier. To determine whether a domestic animal is either a host or a reservoir for *C. cayetanensis*, we first examined feces from cats, which are hosts and reservoirs of *Toxoplasma gondii*, a coccidia causing human illness, but got negative results. Because *Cyclospora* were recently phylogenetically linked to *Eimeria mitis* and *E. tenella* (7), coccidial parasites of chickens, we investigated the presence of *Cyclospora* in poultry.

We pooled feces from approximately 600 4- to 6-week-old chickens from a poultry farm near Monterrey, Mexico, and extracted feces from the caecum of 50 6- to 8-week-old chickens from a poultry market at that location. By Percoll discontinuous-gradient centrifugation (Medina-De la Garza et al., submitted), both fecal pools were positive for coccidia, mainly *Eimeria* species and what we regarded as *C. cayetanensis* oocysts. Presence of *Cyclospora* was confirmed by 1) characteristic morphology and size (8µm to 10µm), 2) positive staining with Kinyoun's acid-fast stain, 3) positive autofluorescence under ultraviolet light, and 4) sporulation of oocysts with formation of sporocysts after a 10-day incubation. All these are diagnostic features of *C. cayetanensis* (8) and to our knowledge are not described for any known poultry coccidia.

On the basis of these findings, we suggest that poultry may serve as a possible source for human infection with *Cyclospora*. Consumption of chicken has been reported in one infected patient in the original description by Ashford (1) and in a patient reported recently by Connor and Shlim (9). Moreover, the only existing report of *C. cayetanensis* found in feces from a domestic farm animal concerned a farm duck (6). Zerpa et al. suggest that besides consumption of contaminated water, other modes of transmission involving contact with domestic animals must be considered. So far, however, a possible infection route involving poultry, whether it may be direct consumption of undercooked chicken meat, contamination of food and water sources with chicken feces, or both, remains to be determined. It should be noted that sanitary standards in poultry-breeding facilities in developing countries may not be adequate. This would account for the fact that reports implicating chickens in the transmission of *Cyclospora* (1,9) have occurred in, or in relation to, developing countries. The *Cyclospora* found in the chickens in our study have the diagnostic features of C. *cayetanensis*. Nevertheless, the existence of another, not yet described, *Cyclospora* species infecting poultry, which has similar features but is different from *C. cayetanensis*, cannot be excluded at this stage. In addition, the number of oocysts recovered was not large and because feces were pooled, we could not calculate the number of oocysts passed by each bird. The possibility that oocysts were acquired as a contaminant from food or water sources and were only passing though the gut of the chickens (making the chickens a paratonic host) cannot be ruled out.

The increased recognition of *Cyclospora* as an important cause of diarrhea in both immunocompromised and immunocompetent persons and the public health relevance of this emerging pathogen as a potential cause of diarrheal outbreaks (3,4) make prompt disclosure of the epidemiologic features and behavior of the parasite necessary. As we propose the possible participation of poultry in the epidemiologic cycle of the coccidia, we invite other *Cyclospora* working groups worldwide to confirm the so far putative reservoir described in this communication and to further study other possible hosts or reservoirs.

**H. Leslie García-López, Luís E. Rodríguez-Tovar, and Carlos E. Medina-De la Garza***
Facultad de Medicina y Hospital Universitario "Dr. J.E. González," Universidad Autónoma de Nuevo León, Monterrey, Mexico

### References

1. Ashford RW. Ocurrence of an undescribed coccidian in man in Papua New Guinea. Ann Trop Med Parasitol 1979;73:497-500.
2. Ortega YR, Gilman RH, Sterling CR. A new coccidian parasite (Apicomplexa: Eimeriidae) from humans. J Parasitol 1994;80:625-9.
3. Soave R, Johnson WD. *Cyclospora:* conquest of an emerging pathogen (commentary). Lancet 1995;345:667-8.
4. Huang P, Weber JT, Sosin DM, Griffin PM, Long EG, Murphy JJ, et al. The first reported outbreak of diarrheal illness associated with *Cyclospora* in the United States. Ann Intern Med 1995;123:409-14.
5. Rabold JG, Hoge CW, Shlim DR, Kefford C, Rajah R, Echeverría P. Cyclospora outbreak associated with chlorinated drinking water. Lancet 1994;344:1360.
6. Zerpa R, Uchima N, Huicho L. *Cyclospora cayetanensis* associated with watery diarrhoea in Peruvian patients. J Trop Med Hyg 1995;98:325-9.
7. Relman DA, Schmidt TM, Gajadhar A, Sogin M, Cross J, Yoder K, et al. Molecular phylogenetic analysis of *Cyclospora*, the human intestinal pathogen, suggests that is closely related to *Eimeria* species. J Infect Dis 1996;173:440-5.
8. Chiodini PL. A "new" parasite: human infection with *Cyclospora cayetanensis*. Trans Roy Soc Trop Med Hyg 1994;88:369-71.
9. Connor BA, Shlim DR. Foodborne transmission of cyclospora . Lancet 1995;346:1634.

### PCR Confirmation of Infection with *Cyclospora cayetanensis*

**To the Editor:** *Cyclospora cayetanensis*, formerly known as cyanobacterium-like body, is a variably acid-fast microorganism. Recently, it was classified as a coccidian parasite (1) closely related to the genus *Eimeria* (2). Humans infected with *C. cayetanensis* typically have diarrheal illness with a variable number of stools per day and sometimes have nausea and vomiting (3,4). *Cyclospora* infection has been reported in many parts of the world as clustered or sporadic cases (1,3-5).

Variable success in diagnosing infection with this parasite underscores the need for using (as quality control) molecular methods, which do not rely on the level of expertise of laboratory personnel in microscopy. The key features for diagnosis by light microscopy are size (8µm to 10µm in diameter), internal features of stained and unstained oocysts, and autofluorescence of oocysts (1,6). The definitive diagnosis is understood as visualization of characteristic sporulated oocysts, which contain two sporocysts. However, sporulation typically requires incubating oocysts for up to 2 weeks, and this approach cannot be applied to Formalin or polyvinylalcohol-preserved stool smears.

Sporadic and clustered cases of *Cyclospora* infections were reported in the United States and Canada during May and June 1996 (5,7). From these outbreaks, more than 900 cases were diagnosed by examining stool specimens under light microscopy (Barbara Herwaldt, pers. comm.). Epidemiologic studies indicated risk for *Cyclospora* infection from consuming raspberries imported from Guatemala (7). Forty-two stool specimens supplied in 2.5% potassium dichromate from patients with intestinal symptoms were forwarded to the Centers for Disease Control and Prevention to be evaluated by microscopy and by polymerase chain reaction (PCR) amplification. In addition, one well-characterized positive stool specimen from Nepal was provided by John Cross, Armed Forces Research Institute of Medical Sciences,

